# Report on Vital Statistics, Virginia State V. M. A.1Read before the Virginia State Veterinary Medical Association, June 18, 1895.

**Published:** 1895-09

**Authors:** E. P. Niles

**Affiliations:** Chairman, Blacksburg, Va.


### REPORT OF THE COMMITTEE ON VITAL STATISTICS.[1]

BY E. P. NILES, D.V.M., CHAIRMAN,
BLACKSBURG, VA.

As chairman of the Committee on Collective Statistics I beg to submit the following report, much of which I have copied word for word from reports of individual members. The work of getting up this report has been left entirely with the chairman of the committee; one member having moved from the State, and the other having his time occupied in preparing for a trip to Europe.

I wish to take this opportunity to thank those of the Society who have kindly responded to the call of the committee, and to urge those who have not to strain a point in that direction in the future. The notes which have been received from various members of the Association are interesting. Many of them are especially valuable and show close observation. Some of the diseases reported are practically new to us, or at least they have not been recognized as such when seen.

One case of acute nephritis is reported in a gelding eight years old, which was stabled in a damp and poorly ventilated basement. The patient was doing fairly well when it got cast in its stall one

[1] Read before the Virginia State Veterinary Medical Association, June 18, 1895.

night and died before morning. Post-mortem revealed a calculus in the substance and pelvis of right kidney. The stone was arranged in distinct layers loosely adherent to each other.

Infectious abortion is reported as existing in five herds of cattle in 1894. The usual preventive measures and treatment were adopted with satisfactory results.

Dr. Drake reports tuberculosis as being prevalent among thoroughbred, especially Jersey, dairy cattle in his section of the country.

The question as to whether or not fistula and poll-evil are contagious is raised in the same report, the doctor having some reason to suspect such to be the case. The same party is desirous of hearing the views of some older members as to what line of treatment, both curative and preventive, is most effectual. The contagiousness of fistula and poll-evil has never been demonstrated, yet there are circumstances which certainly point in that direction in many cases.

We now come to the most important part of our report: the discussion of some diseases which are reported to exist in and around Richmond by our worthy President. Horses taken to the city of Richmond from other States, and especially from the Western States, are reported almost invariably to undergo an attack of influenza, which is considered present at all times. The infection is not confined to the sale stables, but extends to private stables where but one horse is kept. The question naturally arises, Is it not possible that the infective medium is perpetuated and in some way communicated to other horses of the city by the continued arrival of sale-stock from other States? During the past year, however, the disease has been less frequently met with, and in a milder form and less liable to fatal complications than for a number of years past. One complication, or sequel, however, has been noticed to be of greater frequency of late than of former years, intra-ocular inflammation, a description of which we quote: "The complication usually manifests itself after convalesence has commenced, but occasionally during the height of the fever. The extent and nature of this ocular trouble varies in different animals and seldom affects more than one eye. The disease, as a rule, appears as an iritis, often complicated with disease of the lens or its capsule, and later on the cornea becomes involved as well as the conjunctiva." . . . . In many cases the inflammatory exudate apparently consists of pus or blood, or both, appearing as a sedimentary deposit at the bottom of the anterior chamber of the eye. Treatment was satisfactory in most cases, but not all. It will be seen by the description that this complication differs from the usual ocular complication of influenza in that the inflammation begins within the orbit, finally extending to the conjunctiva and other membranes of the eye, while the reverse is true of the usual complication. Locomotor ataxia is also reported as a complication in three cases, two of which made a good recovery; the third is classed as incurable, showing the same symptoms as those generally seen in so-called immobility. The want of muscular co-ordination in this animal is said to be as perceptible in the fore-limbs as the hind ones.

Contagious pleuro-pneumonia of the horse is reported as being confined almost entirely to horses in the sale stables recently shipped to the city from other points. Owing to the unsanitary conditions under which horses are shipped and long distances, the constitution is so weakened that the animal rapidly succumbs to the disease. The disease is usually found in the same stable with influenza, and may easily be mistaken for pulmonary complications of the latter.

Strangles is reported as being more prevalent during the last few months than for several years previously. It is not confined to horses or colts of any particular age or breed. With one exception it has not been met with in the same animal the second time. The following is quoted from the original report: "Strangles still prevails, but to a less extent than in the spring. I had no fatalities from it, but one case worthy of note was a result of it. In February I attended a yearling bay filly; the abscesses were slow in forming and contained but little pus when opened. She recovered from the general effects of the disease, but the submaxillary and subparotid lymphatic glands remained large and indurated. Blistering had no effect on the enlargements, and I resorted to other means. On April 13th, with a hypodermic syringe, I injected tincture of iodine into the enlargements on the right side, and I was about to repeat the injections on the left side when she suddenly fell and became comatose. She was very restless and fought the twitch very much. Hypodermic injections of stimulants, and other means, were tried to rouse her, but she never regained consciousness, and died about 10 P.M., three hours after she fell. Post-mortem examination held next morning a little after daylight revealed abdominal organs normal; both ventricles of heart contained a little frothy, dark blood, but the organ itself was normal; both lungs œdematous, the right one being very much hypostatically congested, having laid and died on the right side; the bronchial tubes were glutted with a frothy mucus; the mucous membrane of trachea and larynx dark from congestion, but showing no signs of recent inflammation. The enlarged glands were carefully dissected, and the tracks of the needle of the hypodermic syringe were easily traced by the iodine discoloration. By the same means it was ascertained that the iodine was confined to the indurated glands, and that neither the needle nor the iodine interfered with any vessel or nerve. There was a small cavity in the gland filled with inspissated pus, which was evidence of the fact that this cavity would have had to be discovered and evacuated before a cure could be effected. The brain was next examined, and immediately on exposure the remarkably blanched appearance of the gray matter pointed out its anæmic condition." In view of the history of the case and the enlarged glands in the region of the carotids, and the anæmic condition of the brain, death was attributed to embolism.

Glanders is reported to exist to some extent in the city of Richmond, but probably limited to a few individual animals. Suspicious cases are occasionally met with, but the symptoms are not sufficiently pronounced to justify destruction. One case of epistaxis is reported in which ulcers were present under the wings of both nostrils. These ulcers, however, lacked the characteristic ragged edges and pit-like appearance of glanders ulcers. Treatment was prescribed and the mallein test recommended, but the owner refused the latter on account of expense. The case is reported as doing well under treatment.

The treatment of atrophy of the scapular muscles by hypodermic injections of solutions of common salt is reported as having been followed by malignant œdema. As the sterilization of the needle of the syringe was the only antiseptic precautions used, it is probable that the germ of the disease was introduced along with the needle. Since the occurrence of this complication, and that of tetanus and abscesses, the doctor has observed strict aseptic rules, such as shaving the hair and scrubbing the skin with creolin, boiling the solution of salt, and thoroughly disinfecting the entire syringe, with the result that not a single complication has followed this line of treatment since. We may add that the efficacy of the free use of antiseptics and other necessary aseptic precautions is demonstrated in the above. The one case of malignant œdema specially referred to, however, may not have been due to the hypodermic injection, as a wound on the shoulder is reported, through which the germ may have entered. On the other hand, the absence of complications, when more strict precautionary methods are pursued in the injections, would point to infection by means of the injection.

No cases of anthrax have been reported, and so far as I am able to learn the disease is almost unknown in the State. In the middle of last summer, however, a farmer living a few miles north of here arranged to flood his pasture from a small creek, as the grass was dying for lack of rain. This was done in the hottest season of the year, and about two weeks afterward I was called to treat a yearling heifer. The animal had been sick two or three days, and, in fact, was somewhat improved when seen by me. Considering all of the circumstances of the case, I gave it as my opinion that the animal was suffering with a mild attack of anthrax, and that it would probably recover, which it did in due time. About two months ago I was called to Dublin to examine a three-year-old steer in excellent condition. I reached the place late at night, and did not see the animal until after death, as it died that night. Next morning held a post-mortem. Peritoneum inflamed and thickened; abdominal cavity filled with serum; kidneys and bladder normal; circumscribed spot in right lung about eight inches in diameter filled with dark blood. Steer was said to have had hemorrhage from the nostrils before death. As my time was limited, in order to catch the train, I did not make as complete an autopsy as I desired to, but gave it as my opinion that the animal died from anthrax. Microscopical examination confirmed my opinion. About two weeks before this animal was taken sick a small stream of water running through the pasture had overflowed, washing a great deal of débris out on the pasture. As the grass grew more rapidly here than on the high ground the cattle naturally grazed this place more than others. I attribute the disease in both instances to the overflow of the pasture.

Enzoötic hematuria, or toxic gastro-enteritis, as a result of feeding upon various kinds of shrubbery, is reported to have affected a number of colts, some of which died before being treated. All that were treated made a good recovery. The following is a copy of the symptoms and post-mortem appearances of the disease: "Animal stood dull and listless, taking little or no notice of what was passing about him; eyes either half or wholly closed, head down, breathing slowly, abdomen tucked up in flanks, giving a gaunt appearance; visible mucous membranes of mouth and eyes icteric. When they moved it was with a staggering gait. One was caught for the purpose of examination and became very excited, and in the struggle fell, still continuing to struggle so much that I decided not to record the pulse or temperature, as under such circumstances they would not have been trustworthy. In age the colts ranged from one to three years. . . . One of the colts (a two-year old) died some time during the night. Autopsy about 1 P. M.: Decomposition had set in; subcutaneous tissue icterical; lungs collapsed; stomach gorged with a coarse, fibrous, somewhat moist, fetid mass of ingesta mixed with grass; cæcum and colon also contained ingesta of the same kind, but not in such large quantities; the dung was soft throughout; inflammation extended through entire digestive tract from stomach to anus; spleen visibly enlarged, but contents not noticeably deranged; liver soft and friable, and of a color impossible to describe, but reminding one of an odd lead-color; kidneys enlarged and inflamed, with collection in pelvis of very thick matter resembling pus; bladder inflamed, and containing a large quantity of bloody urine, at least urine so appeared to the naked eye." It is thought that the above disease is often diagnosed as anthrax when no microscopical examination is made.

Gangrenous dermatitis is reported as affecting a number of horse and mule colts on one farm. Large portions of the skin became gangrenous, and the parts discharged pus profusely. It is supposed that the presence of lice caused the colts to rub until the skin became abraded, allowing the entrance of vegetable parasites, probably moulds, which would seem to be the direct cause of the gangrene. We would suggest that this gangrene may have been caused by the presence of pus-forming bacteria, which, in view of the profuse purulent discharge, must have been present in great numbers. Senn, in his *Principles of Surgery*, describes a form of gangrene caused by the streptococci. He also says: "All bacteria which can produce an inflammation sufficiently severe to completely arrest circulation can become an indirect cause of necrosis." It would, however, require a careful microscopical examination to ascertain whether the gangrene in question was due to a mould or bacteria. Your committee is inclined toward the latter.

Southern cattle fever was prevalent in various parts of the State during the last summer and fall.

Tuberculosis is reported as being prevalent among the dairy herds in the State. Out of one herd of 130 cattle reported in Bulletin No. 7 of the Bureau of Animal Industry, 90 reacted to the tuberculin test. But few of the cattle were destroyed, but those that were showed tuberculous lesions, proving the accuracy of the tuberculin test. This herd is said to have every advantage of good care, fine buildings, and good surroundings.

Canine distemper is reported to have prevailed to a great extent during the past year, with great fatality. The type was principally the nervous form, which gives rise to so many "mad-dog" scares. The malady still exists, and cases are frequently met with. A fine pointer owned by the foreman on the college farm was thought to be "mad" a few days ago, and was at once destroyed before I had opportunity to see him. As described to me, however, the disease was certainly the nervous form of canine distemper; the only case I have heard of in this section.

Dr. Williamson, of North Carolina, reports a form of diarrhœa in colts (gastro-enteritis), which causes severe losses in very warm, wet weather. The disease also extends to young calves. It is, however, successfully treated with decoctions of oak-bark, preferably the bark of red oak. One ounce of the decoction is given three times daily, or oftener if necessary.

During the month of February a disease made its appearance which at first was thought to be only a variation of influenza, and other cases of it were treated for strangles, as the latter disease was prevalent at the time. The owners of several infected animals told me that they had had colt distemper when they were younger, and then the number of cases not having abscesses led me to watch the primary symptoms with closer attention; these points, in connection with the fact that the usual symptoms of influenza, such as debility, ophthalmia, anorexia, extremely high temperature, lung and pleural complications, etc., were wanting, led me to the conclusion that I had to deal with an independent disease which was not a complication of either strangles or influenza. At first I did not consider the disease infectious, as but one or probably two horses would be affected in a large stable. The weather, which was very changeable, often wet and cold, was thought to be sufficient cause, but after a time instances frequently occurred that caused me to change my mind, and I now think the affection an infectious one, though mild. About the first symptom noticed is difficulty in swallowing; the animal, although having a desire for food and water, abstains from both on account of the pain caused by the attempts to swallow. Water is returned through the nostrils; also food, which is especially noticeable, when the animals have been eating grass, by the green color of the discharge. In nearly every case one or both thyroid glands are enlarged. The sublingual, submaxillary, and subparotid lymphatics become enlarged and painful; occasionally the parotid glands are involved. The temperature may rise to 105° F., but it is usually much less. If debility occurs it is due to inability to swallow food, and not the effect of fever or the constitution. Flesh is not noticeably lost except in the cases where the difficulty in swallowing lasts for some days. The appetite is not lost, nor is the desire for water. Pressure over the region of the pharynx causes much pain and is violently resisted. After two, three, or four days a discharge of pus from the nostrils appears, which is soon followed by improvement in the act of swallowing. In a few days the enlarged glands begin to assume their normal condition. The majority of the cases recover in about ten days, showing little if any bad effects of the attack. Occasionally an abscess will form in the intermaxillary region, but never in connection with the thyroids. Occasionally a case is met with where the breathing is difficult and noisy for a day or two, which is most probably due to a larger abscess than usual in the pharynx interfering with the larynx, but as soon as a profuse discharge of thick yellowish pus appears from the nostrils, showing that the abscess has burst, the breathing becomes easier. The cough is made much worse by attempts to swallow food. After the discharge appears, large lumps and flakes are coughed up or dislodged by the cough and discharged from the nostrils There were no fatalities and no sequalæ. All cases made satisfactory recoveries, most of them in a comparatively short time, but a few remained on the list for a much longer time than the average. Sporadic pharyngitis is by no means an uncommon affection, but we meet with it in isolated instances, and we know that the first stage of regular strangles is a pharyngitis, but this is my first experience with pharyngitis as an enzoötic, or as it may be called an epizoötic, as the cases were not confined to Richmond; I had quite a number of cases in Manchester and in the suburbs of both cities. I did not see a case in a mule.

The most cases were met with in April and May, and I still see a case occasionally.

Two other interesting cases have been reported to your committee, but, as they are surgical cases, it was thought best to bring them up for discussion under the head of special cases.

Attention is called to the fact that no law exists in the United States looking to the prevention and control of such diseases as influenza and strangles. It is, nevertheless, a fact that these diseases cause the loss of many animals, and are spread by the loose manner in which horses are allowed to be shipped. There is a law purporting to control glanders in this State, but it is w orse than none, having been framed and passed by parties who evidently did not know that such a being as a veterinary surgeon existed.

In conclusion, your committee respectfully urges each member of the Association ever to be on the lookout for interesting notes on the various diseases, whether common or not, which may come under their observation. To that portion of the medical profession who have joined us, we beg to suggest that they turn a portion of their attention to the collection of such notes as would be of interest from a comparative standpoint.

